# Giant splenic artery aneurysm rupture mimicking a pancreatic pseudocyst: a diagnostic pitfall on non-contrast computed tomography

**DOI:** 10.1093/jscr/rjag440

**Published:** 2026-06-13

**Authors:** Zhi-Ping Zhang

**Affiliations:** Emergency Department, Wuhan Asia General Hospital, 300 TaZi Lake North Road, Wuhan 430056, China

**Keywords:** splenic artery aneurysm, rupture, hemoperitoneum, shock, CT angiography

## Abstract

Splenic artery aneurysm (SAA) rupture is a rare but life-threatening cause of acute abdominal pain, with mortality exceeding 25% in undiagnosed cases. This report describes a giant SAA rupture that was initially misdiagnosed as a pancreatic pseudocyst on non-contrast computed tomography (CT), highlighting a critical diagnostic pitfall. A 49-year-old hypertensive male presented with sudden-onset left upper quadrant pain and nausea. Initial non-contrast CT showed cystic lesions with peripheral calcification, which were radiologically interpreted as a pancreatic pseudocyst. The patient subsequently developed hemodynamic collapse due to a “double rupture” phenomenon. Emergency CT angiography revealed a ruptured giant SAA (5.2 cm). Emergency laparotomy and splenectomy were performed successfully. This case illustrates the limitations of non-contrast CT in differentiating SAAs from cystic pancreatic lesions and reinforces the necessity of a low threshold for CT angiography in unexplained upper abdominal pain.

## Introduction

The splenic artery accounts for ~60% of all visceral artery aneurysms, with a prevalence ranging from 0.7% to 10% in autopsy studies [1]. While the majority of splenic artery aneurysms (SAAs) remain asymptomatic, rupture is a catastrophic complication associated with a mortality rate of 10% to 25% [2]. “Giant” SAAs, typically defined as those with a diameter greater than 2 cm, represent a high-risk subgroup with a significantly increased potential for spontaneous rupture [3].

The clinical presentation of SAA rupture is often non-specific. The classic triad of left upper quadrant (LUQ) pain, Kehr’s sign, and hypovolemic shock is absent in up to 40% of patients [4]. Consequently, clinicians rely heavily on imaging. However, on non-contrast computed tomography (CT), a giant SAA with peripheral calcification can closely mimic benign cystic entities, such as pancreatic pseudocysts or cystic neoplasms. This diagnostic ambiguity frequently leads to delayed intervention. We present a case of a “near-miss” diagnosis where a ruptured giant SAA was initially misidentified as a pancreatic lesion on non-contrast CT. This report aims to emphasize the limitations of non-contrast imaging and the critical importance of early contrast-enhanced CT angiography (CTA) in preventing fatal diagnostic delays.

## Case report

A 49-year-old male with a medical history of hypertension presented to the emergency department with a 3-h history of sudden-onset, persistent dull pain localized to the LUQ, associated with nausea. He denied fever, trauma, or changes in bowel habits. On admission, his vital signs were stable: blood pressure 123/75 mmHg, heart rate 78 beats/min, respiratory rate 18 breaths/min, and body temperature 36.5°C. Physical examination revealed mild deep tenderness in the LUQ without signs of peritonitis. Initial laboratory investigations, including a complete blood count and serum amylase levels, were within normal limits.

An urgent non-contrast abdominal CT scan demonstrated two round, cystic, low-density lesions with peripheral calcification in the left upper abdomen, adjacent to the pancreatic tail (Fig. 1). The larger lesion measured 52 × 44 mm. Peripancreatic fat stranding obscured the pancreatic contours, and the radiological report initially suggested a “differential diagnosis of pancreatic pseudocyst or cystic neoplasm.” No definitive vascular pathology was identified at that time, and the patient was admitted for observation.

**Figure 1:**
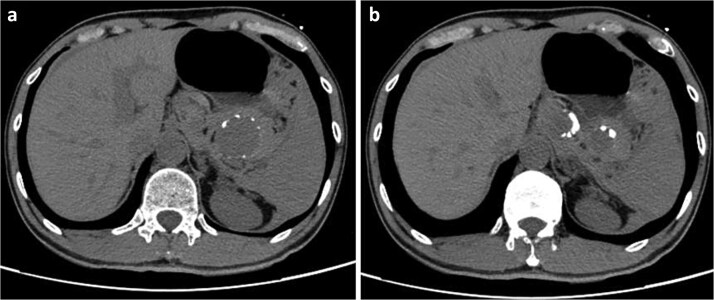
Initial non-contrast axial abdominal CT scan. (a) Two round cystic low-density lesions with peripheral calcification in the left upper abdomen. (b) The larger lesion measuring 52 × 44 mm adjacent to the pancreatic tail.

Approximately 6 h after admission, the patient experienced an abrupt clinical deterioration, presenting with agitation, diaphoresis, and profound hypotension (60/40 mmHg) accompanied by tachycardia. Emergency contrast-enhanced CTA was immediately performed. The CTA revealed two round, nodular, high-density lesions in the mid-distal splenic artery, measuring 36.7 × 27.3 mm and 42 × 52.7 mm, respectively (Fig. 2). Active extravasation of contrast material from the aneurysm sac was noted, with multiple surrounding patchy isodense opacities consistent with acute hemoperitoneum (Fig. 2).

**Figure 2:**
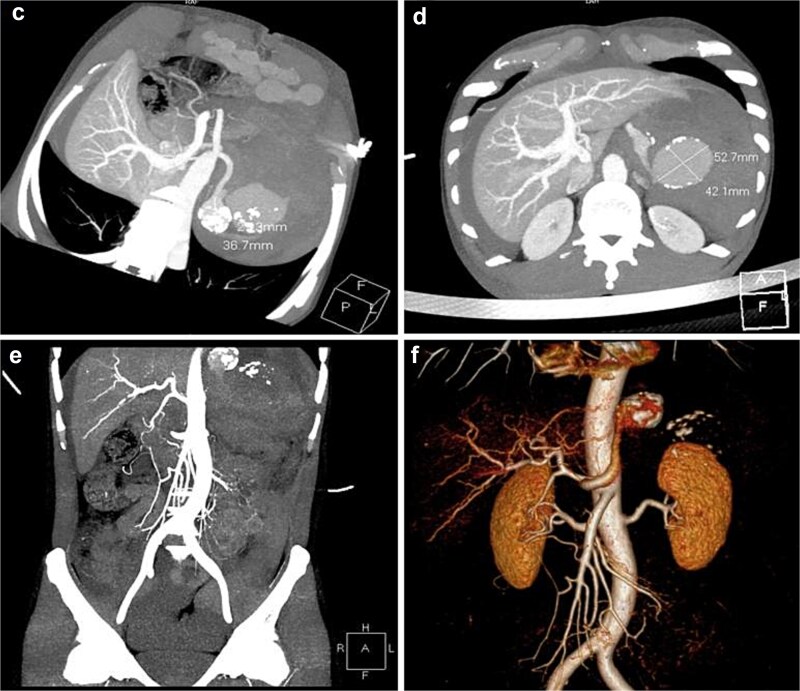
Emergency contrast-enhanced CTA. (c and d) 3D reconstruction and axial imaging reveal two round nodular high-density lesions in the distal splenic artery, measuring 36.7 × 27.3 mm and 42 × 52.7 mm, respectively. (e and f) Active extravasation of contrast material and surrounding patchy opacities consistent with acute hemoperitoneum.

A diagnosis of ruptured giant SAA with hemorrhagic shock was established, and the patient was rushed for an emergency exploratory laparotomy. Intraoperatively, 3000 ml of blood and clots were evacuated from the intraperitoneal cavity. A ruptured bilobed aneurysm at the splenic hilum was identified as the source of hemorrhage. Definitive surgical management included splenectomy and en-bloc aneurysm resection.

Histopathological examination confirmed a true saccular aneurysm with atherosclerotic changes and medial wall hypoplasia. Gross examination revealed dark, irregular aneurysmal tissue measuring ~5–7 cm in diameter (Fig. 3). Microscopic analysis demonstrated arterial wall thinning with focal disruption and an adjacent organizing hematoma (Fig. 3). The patient’s postoperative course was managed in the intensive care unit, and he recovered uneventfully. He was discharged on postoperative day 12.

**Figure 3:**
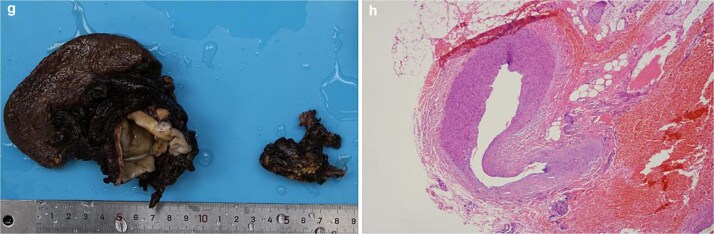
Pathological findings. (g) Gross specimen showing dark, irregular aneurysmal tissue with internal cystic spaces, measuring 5–7 cm. (h) Photomicrograph (H&E stain) demonstrating thinned arterial wall with focal disruption and adjacent organizing hematoma.

Written informed consent was obtained from the patient for the publication of this case report and accompanying images, in accordance with the Declaration of Helsinki.

## Discussion

This case highlights a critical diagnostic challenge in emergency surgical practice, emphasizing the significant limitations of non-contrast CT for detecting vascular emergencies. The initial imaging finding of a peripherally calcified cystic mass can mimic benign entities such as pancreatic pseudocysts or cystic neoplasms [4]. Without intravenous contrast, a thrombosed or partially thrombosed SAA may be indistinguishable from these conditions, leading to dangerous delays in treatment.

A key clinical feature discussed in this context is the “double rupture” phenomenon. In this scenario, the initial hemorrhage is contained within the lesser sac, providing a period of temporary clinical stability that may last from hours to days. However, as the pressure within the lesser sac increases, blood eventually bursts through the foramen of Winslow into the general peritoneal cavity, resulting in sudden hemodynamic collapse and hypovolemic shock. This sequence was clearly observed in our patient, who deteriorated abruptly ~6 h after an initially stable admission.

The patient presented with a “giant” SAA, which in this instance exceeded in diameter. While the established size threshold for prophylactic intervention in asymptomatic SAAs is generally or larger, giant aneurysms carry a markedly increased risk of spontaneous rupture [5, 6]. Notably, this patient lacked classic risk factors such as portal hypertension, pregnancy, or prior liver transplantation, emphasizing that SAA rupture can occur in seemingly low-risk individuals. Histopathological examination revealed medial wall hypoplasia and poor arterial development, indicating a potential underlying structural weakness that predisposed the vessel to aneurysmal formation and subsequent rupture.

In terms of management, emergency open surgery remains the mainstay of treatment for ruptured SAAs associated with hemodynamic instability, as it allows for rapid source control and evacuation of hemoperitoneum [7]. While endovascular techniques, such as coil embolization, represent viable minimally invasive alternatives for stable patients or elective settings [8], the rapid clinical decline in this case necessitated an immediate surgical approach.

## Conclusion

Ruptured giant SAA illustrates a critical diagnostic pitfall in emergency medicine due to the limitations of non-contrast CT in identifying vascular pathology. This case reinforces the importance of maintaining a high index of suspicion for vascular emergencies in patients presenting with unexplained acute abdominal pain. We advocate for the prompt utilization of contrast-enhanced CTA as the definitive diagnostic tool in such scenarios, as it enables direct visualization of the vascular anatomy and guides immediate, life-saving intervention.
